# Enhanced Recovery after Surgery in a Single High-Volume Surgical Oncology Unit: Details Matter

**DOI:** 10.1155/2016/6830260

**Published:** 2016-08-25

**Authors:** Timothy L. Fitzgerald, Catalina Mosquera, Nicholas J. Koutlas, Nasreen A. Vohra, Kimberly V. Edwards, Emmanuel E. Zervos

**Affiliations:** ^1^East Carolina University Brody School of Medicine, Division of Surgical Oncology, Greenville, NC 27834, USA; ^2^East Carolina University Brody School of Medicine, Department of Surgery, Greenville, NC 27834, USA; ^3^Vidant Medical Centre, Division of Dietetics and Nutrition, Greenville, NC 27834, USA

## Abstract

Benefits of ERAS protocol have been well documented; however, it is unclear whether the improvement stems from the protocol or shifts in expectations. Interdisciplinary educational seminars were conducted for all health professionals. However, one test surgeon adopted the protocol. 394 patients undergoing elective abdominal surgery from June 2013 to April 2015 with a median age of 63 years were included. The implementation of ERAS protocol resulted in a decrease in the length of stay (LOS) and mortality, whereas the difference in cost was found to be insignificant. For the test surgeon, ERAS was associated with decreased LOS, cost, and mortality. For the control providers, the LOS, cost, mortality, readmission rates, and complications remained similar both before and after the implementation of ERAS. An ERAS protocol on the single high-volume surgical unit decreased the cost, LOS, and mortality.

## 1. Introduction

The concept of enhanced recovery after surgery (ERAS) involves the integration of evidence-based medicine into clinical practice to improve patient outcomes. The application of new evidence is often delayed and inconsistently adapted into clinical care [1–3]. The genesis of ERAS is associated with the colonic surgeries of the 1990s and is often attributed to Professor Henrik Kehlet of Copenhagen [4]. Kehlet reported length of stays (LOS) of 2-3 days for patients undergoing colonic surgery in an era when LOS was typically more than 9 days. The term ERAS was coined in 2001 by a group of academic surgeons in Europe working on protocols for colon surgery [5], who were heavily influenced by Kehlet's remarkable results.

The basic tenets of ERAS include the adoption of evidence-based practices to decrease surgical stress, maintain physiologic homeostasis, and facilitate recovery of patients [6, 7]. Although individual components may vary, most of the ERAS programs include avoidance of fasting, optimization of health, preoperative carbohydrate loading, avoidance of bowel preparation, goal-directed resuscitation, multimodal analgesia with avoidance of opiates, avoidance/early removal of tubes (nasogastric tube, Foley catheter, and drains), support of gastrointestinal function, and early convalescence [4, 7, 8]. When these principles are applied to the patients undergoing colonic surgery, factors such as LOS, complications, and readmission rates are noted to have decreased [8–10]. Improved outcomes with ERAS implementation in colon surgery have been documented in randomized controlled trials and structured nationwide programs [11, 12].

The majority of data on ERAS focus on colon surgeries, and its success has encouraged expansion to other major abdominal operations. In a study, Joliat et al. noted a decrease in LOS and cost after pancreaticoduodenectomy with an ERAS program [13]. In a systematic review and meta-analysis of nine trials (two were randomized controlled), Hughes et al. reported that ERAS protocols were associated with decreased morbidity and LOS after hepatic resection [14]. Similar results have been reported in cases of bladder, esophagus, gastric, bariatric, gynecologic, and emergent surgeries that have implemented the ERAS protocols [15–25].

To better understand the application of ERAS protocol in a diverse patient population and the influence of shifting expectations on non-ERAS patients, we reviewed a pilot program involving a single gastrointestinal oncology surgeon on a closed surgical oncology unit. Our hypothesis was that a single protocol encompassing the tenets of ERAS when applied to a myriad of complex abdominal procedures would improve the patient outcomes. In addition, we also hypothesized that education of perioperative staff and changes in the postoperative expectations would improve the outcomes in the unit's control (non-ERAS) population.

## 2. Methods

### 2.1. Data Source

Patients undergoing only elective abdominal surgeries from June 2013 until April 2015 at Vidant Medical Center, East Carolina University, Greenville, North Carolina, were included in this study. Patients of three attending surgical oncologists were retrospectively identified from the University Health Consortium (UHC) database using the primary procedure codes. Patients who underwent emergent or urgent procedures were excluded. All patients undergoing minor nonabdominal or abdominal procedures were also excluded. The excluded abdominal procedures included soft tissue excision, open liver biopsy, closed liver biopsy, and excision of lymph nodes. The demographics, operative factors, and weighted financial data of the patients were collected. The electronic health record was utilized for data that was unclear in the UHC dataset or was unavailable. The age-adjusted Charlson Comorbidity Index (CCI) was calculated for each patient using comorbidities within the UHC dataset and ICD-9 codes. Then the patients were divided into three subcategories based on their CCI score: low, with CCI scores of 1-2; intermediate, with CCI scores of 3–5; and high, with CCI scores of >5. The surgical procedures were divided into six subcategories: pancreatectomy, intestinal resection, hepatic resection, colostomy, gastrectomy, and other major abdominal surgeries. The LOS, in-hospital mortality, readmission within 30 days, and postoperative complications were recorded. The Clavien-Dindo score was utilized to classify postoperative complications. All costs during the index admission period were calculated using the adjusted financial data within the UHC dataset.

### 2.2. Implementation of Protocol

A protocol was developed that utilized the published guidelines by the ERAS Society for pancreatic, colonic, and rectal/pelvic surgeries [26, 27]. The goal was to develop a single perioperative process that could be applied to a diverse group of patients. Protocol components included preoperative immunonutrition, clear liquids until 2 hours before surgery, carbohydrate loading until 2-3 hours before surgery, epidural catheter offered to all patients undergoing laparotomy, loading dose of gabapentin, intraoperative goal-directed resuscitation with LIDCO monitor (LIDCO Ltd., London, UK), maintenance of normothermia, multimodal pain management with avoidance of narcotics, avoidance of nasogastric tubes, early removal of drains, perioperative bowel regiment, postoperative immunonutrition, and early aggressive convalescence (Table 1).

As controversies existed around the impact of enhanced recovery programs versus shifting expectations, patients from a single health care provider were used for tests of change; however, educational interventions included all the staff members. Educational interventions targeted anesthesiologists and anesthetists, dieticians, postoperative nursing staffs, preoperative nursing staffs, and surgical residents. Multiple educational conferences were delivered to the aforementioned key stakeholder groups involved in the health care program for patients at a single and closed surgical oncology unit. In addition, the surgeon, clinical nursing staffs, and a registered dietitian performed patient education during the preoperative visit. Patients were also provided with written instructions. The ERAS protocol was implemented on the patients from a single provider in June 2014 (test surgeon) whereas the patients from other health care providers served as the internal control individuals.

Logistic regression was undertaken to determine differences between patients included in the ERAS protocol (test surgeon after 2014) versus those managed without ERAS (test surgeon before 2014 and control surgeons). ERAS outcomes were also analyzed for the test surgeon, comparing patients before and after protocol implementation. Lastly differences in outcomes were evaluated for the control surgeons before and after implementation of protocol by the test surgeon in 2014.

### 2.3. Statistical Analysis

Patient demographics, in addition to their operative and financial variables, were represented as means or medians as necessary. Student's *t*-test and chi-square test were performed for univariate analysis. Variables, having a *p* value < 0.2 in univariate analysis, were included in all multivariate models. A *p* value < 0.05 was defined as statistically significant. The analysis was conducted by using JMP Pro Version 10.0.0 (SAS Institute Incorporated, Cary, North Carolina, USA, 2012).

## 3. Results

### 3.1. Demographics

A total of 394 patients met the inclusion criteria and were included in this study. The mean age of the included patients was 62.3 years (Table 2). The patients were evenly distributed between genders, and white was found to be the most common racial category (59.9%). A majority of the patients were diagnosed with colorectal, pancreatic, hepatic (primary or secondary), gastric, or small bowel cancer. In addition, most of the patients had low (40.4%) or intermediate (39.6%) CCI scores. The most common surgical procedure was other abdominal procedure followed by pancreatectomy, intestinal resection, colectomy, hepatic resection, and gastrectomy surgeries. Two surgeons, the test surgeon and a second surgical oncologist, performed a majority of the procedures. The mean and median values of LOS were 6 and 7.6 days, respectively, and the median hospital cost was $20,998. The in-hospital mortality rate was 2.3%.

### 3.2. ERAS versus Non-ERAS

In order to understand the implications of the ERAS program, we divided the patients into ERAS and non-ERAS groups (Table 3). We found that patients were similar in gender, age, race, diagnosis, comorbidities, and surgical procedures in both groups. There was a decrease of 2 days in mean LOS (*p* = 0.016). The in-hospital mortality was also significantly lower in the ERAS group (0 versus 2.9%, *p* = 0.033). Cost ($18,716 versus $21,294) and readmission rates (16 versus 11.5%) were higher for non-ERAS patients, but these differences did not reach statistical significance (*p* = 0.60 and 0.21, resp.). The complication rates were similar.

## 4. Tests of Change in Single Provider

### 4.1. Enhanced Recovery versus Standard Care

Pre- and post-ERAS outcomes were studied for the test provider (Table 4). Patients in both the groups were similar in gender, age, race, diagnosis, and comorbidities. Mean LOS was significantly decreased during the post-ERAS implementation phase (9.6 days pre-ERAS versus 6.2 days post-ERAS, *p* = 0.024). Costs were also significantly different ($21,674 versus $30,380; *p* = 0.029). The in-hospital mortality rate was also lower in the post-ERAS phase (0 versus 3.3%, *p* = 0.044). The increase in the number of pancreatectomies, major complications, and readmission rates failed to reach statistical significance.

### 4.2. Impact of Changing Culture on Nonparticipating Surgeons

In order to validate the hypothesis that changes after ERAS protocol implementation on this closed, surgical oncology unit were different from the changes in unit culture and expectations, we examined the nontest physicians' patients before and after the educational programs and ERAS implementation. We found that the patients were similar in gender, age, race, diagnosis, comorbidities, surgical procedures, postoperative LOS, complications, median costs, readmission rate, and mortality.  Table 5 shows that the variables before and after the implementation of ERAS in nonparticipating physicians are remarkably similar. This implies that the changes in the patient outcomes are not dependent upon the cultural shifts in patterns of care of the surgical unit.

## 5. Conclusion

ERAS is a multimodal, evidence-based perioperative pathway that has been demonstrated to decrease the cost, LOS, morbidity, and mortality after major surgical interventions. Surgical literature is replete with data describing the outcomes of ERAS implementation in defined patient populations such as colorectal, hepatopancreatobiliary, esophageal, gynecologic, gastric, acute-care, or urologic surgeries [4, 14, 15, 19, 25, 28, 29]. However, there is a lack of data on the applicability of ERAS to real-world diverse surgical practices. It is also unclear whether the differences in outcomes are attributable to the changes in practice or changes in expectations.

In this study, we found that the principles of ERAS could be applied to various abdominal procedures by utilizing a single perioperative pathway. The utilization of this general ERAS pathway for abdominal procedures, including pancreatectomy, hepatectomy, colectomy, gastrectomy, and small bowel resection, resulted in significant decrease in LOS, cost, and mortality rates. Despite extensive training to nursing staffs of the single and closed surgical oncology unit, there were no changes in outcomes in the unit of non-ERAS (control) patients.

To have maximal impact of ERAS protocol, it must be translatable into settings in which most surgeons practice. Majority of general surgeons do not focus on a specific surgery but rather treat patients with a variety of surgical conditions. In this setting, multiple or individual ERAS pathways for each procedure type may not be practical. Our data also suggested that a more general perioperative pathway would suffice to encompass the principles of ERAS. The benefits analyzed by utilizing a single set of preoperative instructions, generalized recommendations for perioperative nutrition, and a single postoperative order set were similar to those reported from more focused programs [8, 14, 15, 19, 27, 28, 30]. The improvements in outcome measures were most marked for procedures that did not have existing postoperative order sets such as other abdominal, hepatectomy, and gastrectomy compared to pancreatectomy and colectomy procedures that had preexisting postoperative pathways (Table 6).

ERAS perioperative pathways have been demonstrated to decrease the cost of care. When ERAS and non-ERAS patients were compared in this series, we found a marked decrease in the costs of approximately $2,500 per patient. Decrease in costs was more in the case of the test surgeon's patients, which was >$5,000 per patient. Although these differences did not reach statistical significance, they are in line with those results reported by other investigators. Joliat et al. noted a decrease of 8000 Euros in costs for patients undergoing pancreatectomy procedure after ERAS implementation [13]. Green reported a $4,800 decrease in costs for patients undergoing colon surgery [31]. Similar results have been also reported in both North American and European series for gynecologic, bariatric, general, and vascular surgeries [32]. Decreasing costs are likely to result from the shorter LOS and fewer complications.

Evidence on decreased LOS and cost due to the implementation of ERAS protocol after major surgical interventions is compelling. In this study, we noted a marked decrease in LOS approximately by three days. Recent meta-analyses have demonstrated a similar decrease in LOS by 2-3 days after colectomy, 2.5 days after hepatic resection, 1.5 days after gynecologic surgery, and 2–6 days after pancreatectomy surgeries [14, 17, 29].

When implementing an ERAS protocol or any other quality program, it is imperative to examine the pre- and post-ERAS implementation data to ensure that any unintended consequences are appropriately detected, and the hypothesized improvements actually occurred in patient outcomes. In this study, we also found a decreased risk of mortality rate in the ERAS cohort (0 versus 3%, *p* = 0.033). Similarly, we found no increase in the readmission rate, which suggested a trend of potential decrease. These findings were in contrast to most of the reports in which ERAS neither increased nor decreased the readmission or mortality rates [17, 18, 33, 34].

The benefits of ERAS cannot be solely attributed to the changes in the provider's education and recovery expectations. In this study, we found that the improvements in cost, LOS, mortality, and readmission rate were confined to the patients enrolled only in the ERAS protocol. This occurred even though all patients emanated from a single specialty clinic and managed in a single closed surgical unit with the same residents, nursing staffs, attending physicians, and allied health staffs, all of whom were educated regarding the ERAS protocol. This suggests the details matter and that is not ethereal shifts in expectations the drive improvement. This is consistent with several prospective randomized controlled trials examining the impact of ERAS protocol implementation on patient outcomes [14, 35–37]. In addition, several investigators have also reported on the improved outcomes having higher compliance with ERAS components [5, 37, 38].

In conclusion, ERAS perioperative protocols improve the patient outcomes. Our data collected in context with the current literature demonstrate that the ERAS protocols can be implemented on a wide range of patients undergoing complex abdominal surgeries. Such protocols need not be procedure specific but should incorporate the general principles of ERAS in a single perioperative program. The benefits from such programs include decreased LOS, cost, and mortality rates. It is our hope that these data would demystify the ERAS implementation for the general surgeons and promote its adoption in mixed surgical practices.

## Figures and Tables

**Table 1:** ERAS protocol.

*Preoperative factors*		
High protein diet	Starting at appointment date	Up to 1 gr/kg, protein/day
Immunonutrition	Five days prior to surgery	Ensure complete liquid 1 can BID, Juben power BID
Clear liquids only	After midnight on day prior to surgery	Gatorade lemon-lime 20 Oz, no cream, no red drinks
Last intake	Three hours prior to surgery	Gatorade lemon-lime 20 Oz

*Intraoperative factors*		
Pain control	Throughout the case	Epidural (optional), gabapentin 600 mg once
Normothermia	Throughout the case	Bair Huger
Fluid resuscitation	Throughout the case	Lidco monitor

*Postoperative factors*		
Pain control	Throughout postoperative time	Avoid narcotic use, gabapentin 600 mg PO q 8 hrs × 3, Toradol 15 mg IV q 6 hrs × 4, Tylenol 1,000 iv as needed
Bowel regimen	Until return of bowel function	Colace 100 mg PO q 12 hrs, Dulcolax suppository 10 mg PR q 24 hrs
Diet	Early enteral nutrition	Immunonutrition × 5 days, diet as tolerated on POD1
Early convalescence	Postop day 0	Up to chair 6–8 hrs, ambulation in the halls 5 times a day
Drain management	Postop days 0-1	NGT removed on postoperative days 0-1

**Table 2:** Demographics for all patients on a single surgical unit, 2013–2015.

Factor	Percent	Number
Gender		
Male	48.2	190
Female	51.8	204

Age		
Median	63	20–93
Mean	62.5	12.9

Race		
White	59.9	236
Black	36.5	144
Other	3.6	14

Diagnosis		
Other abdominal	33.0	127
Colorectal cancer	28.1	108
Pancreatic cancer	15.8	61
Liver malignancies	10.1	39
Gastric cancer	6.5	25
Small bowel cancer	6.5	25

Charlson index		
0–2 (low)	40.4	159
3–5 (intermediate)	39.6	156
>5 (high)	20.0	79

Surgery		
Other abdominal	24.1	95
Pancreatectomy	21.1	83
Intestinal resection	18.3	72
Hepatic resection	13.7	54
Colectomy	17.0	67
Gastrectomy	5.8	23

Provider		
Test physician	45.4	179
Control physician 1	44.9	177
Control physician 2	9.6	38

ERAS		
Yes	22.1	87
No	77.9	307

LOS		
Median	6	1–55
Mean	7.6	6.9

Cost		
Median	20,998	6,052–174,537
Mean	25,040	18,700

Complications		
Grade 0-I	63.2	249
Grade II–V	36.8	145

Readmissions	15.7	61

Mortality rate	2.3	9

**Table 3:** ERAS versus non-ERAS (control) patients on a single surgical unit, 2013–2015.

Factor	ERAS% (number)	Non-ERAS% (number)	*p* value
Gender			
Male	46.0 (40)	48.9 (150)	0.63
Female	54.0 (47)	51.1 (157)	

Age			
Median	62 (20–93)	63 (22–88)	0.36
Mean	61.4 (13.9)	62.8 (12.6)	

Race			
White	62.1 (54)	59.3 (182)	0.89
Black	34.5 (30)	37.1 (114)	
Other	3.5 (3)	3.6 (11)	

Diagnosis			
Gastric cancer	4.8 (4)	7.0 (21)	0.45
Small bowel cancer	8.4 (7)	6.0 (18)	
Colorectal cancer	24.1 (20)	29.1 (88)	
Pancreatic cancer	21.7 (18)	14.2 (43)	
Primary and secondary liver malignancies	12.1 (10)	9.6 (29)	
Other abdominal	28.9 (24)	34.1 (103)	

Charlson index			
0–2	43.7 (38)	39.4 (121)	0.76
3–5	36.8 (32)	40.4 (124)	
>5	19.5 (17)	20.2 (62)	

Surgery			
Pancreatectomy	27.6 (24)	19.2 (59)	0.49
Colectomy	12.6 (11)	18.2 (56)	
Hepatic resection	12.6 (11)	14.0 (43)	
Gastrectomy	4.6 (4)	6.2 (19)	
Intestinal resection	16.1 (14)	18.9 (58)	
Other abdominal	26.4 (23)	23.5 (72)	

LOS			
Median	5 (1–39)	6 (1–55)	0.016
Mean	6.0 (4.9)	8.0 (7.3)	

Complications			
Grade 0-I	67.8 (59)	61.9 (190)	0.31
Grade II–V	32.2 (28)	38.1 (117)	

Cost			
Median	$18,716 ($7,937–$93,804)	$21,294 ($6,032–$174,537)	0.060
Mean	$21,674 ($12,118)	$25,994 ($20,092)	

Readmissions			
Readmission rate	11.5 (10)	16.9 (51)	0.21

Mortality			
Mortality rate	0 (0)	2.9 (9)	0.033

**Table 4:** ERAS versus non-ERAS (control) for test surgeon, 2013–2015.

Factor	ERAS% (number)	No ERAS% (number)	*p* value	OR	*p* value
Gender					
Male	46.0 (40)	46.7 (43)	0.92		
Female	54.0 (47)	53.3 (49)			

Age					
Median	62 (20–93)	63 (28–88)	0.41		
Mean	61.4 (13.9)	63.1 (13.9)			

Race					
White	62.1 (54)	60.9 (56)	0.95		
Black	34.5 (30)	34.8 (32)			
Other	3.4 (3)	4.3 (4)			

Diagnosis					
Gastric cancer	4.8 (4)	12.0 (11)	0.35		
Small bowel cancer	8.4 (7)	5.4 (5)			
Colorectal cancer	24.1 (20)	26.1 (24)			
Pancreatic cancer	21.7 (18)	13.0 (12)			
Liver malignancies	12.1 (10)	15.2 (14)			
Other abdominal	28.9 (24)	28.3 (26)			

Charlson index					
0–2	43.7 (38)	47.8 (44)	0.46		
3–5	36.8 (32)	28.3 (26)			
>5	19.5 (17)	23.9 (22)			

Surgery					
Pancreatectomy	27.6 (24)	14.1 (13)	0.064	2.07	0.069
Colectomy	12.6 (11)	12.0 (11)		Referent	Referent
Hepatic resection	12.6 (11)	20.7 (19)		1.30	0.58
Gastrectomy	4.6 (4)	12.0 (11)		1.07	0.91
Intestinal resection	16.1 (14)	21.7 (20)		1.23	0.64
Other abdominal	26.4 (23)	19.5 (18)		1.63	0.23

LOS					
Median	5 (1–39)	6 (1–55)	0.024		
Mean	6.2 (4.9)	9.6 (9.3)			

Complications					
Grade 0-I	67.8 (59)	54.4 (50)	0.064		
Grade II–V	32.2 (28)	42.6 (42)			

Cost					
Median	18,716 (7,937–93,804)	24,395 (6,052–174,536)	0.029		
Mean	21,674 (12,118)	30,380 (25,723)			

Readmission	11.5 (10)	21.4 (19)	0.076		

Mortality	0 (0)	3.3 (3)	0.044		

**Table 5:** Control providers before and after ERAS implementation, 2013–2015.

Factor	Pre-ERAS	Post-ERAS	*p* value
Gender			
Male	51.3 (61)	47.9 (46)	0.63
Female	48.7 (58)	52.1 (50)	

Age			
Median	63 (28–88)	65 (22–86)	0.78
Mean	62.9 (12.1)	62.4 (12.2)	

Race			
White	59.7 (71)	57.3 (55)	0.93
Black	37.0 (44)	39.6 (38)	
Other	3.4 (4)	3.1 (3)	

Diagnosis			
Gastric cancer	2.5 (3)	7.7 (7)	0.43
Small bowel cancer	7.6 (9)	4.4 (4)	
Colorectal cancer	33.6 (40)	26.4 (24)	
Pancreatic cancer	14.3 (17)	15.4 (14)	
Liver malignancies	6.7 (8)	7.7 (7)	
Other abdominal	35.3 (42)	38.5 (35)	

Charlson index			
0–2	34.5 (41)	37.5 (36)	0.36
3–5	49.6 (59)	40.6 (39)	
>5	16.0 (19)	21.9 (21)	

Surgery			
Pancreatectomy	19.3 (23)	24.0 (23)	0.51
Colectomy	21.9 (26)	19.8 (19)	
Hepatic resection	11.8 (14)	10.4 (10)	
Gastrectomy	1.7 (2)	6.3 (6)	
Intestinal resection	19.3 (23)	15.6 (15)	
Other abdominal	26.1 (31)	24.0 (23)	

LOS			
Median	6 (1–45)	5 (1–44)	0.51
Mean	7.6 (5.9)	7.1 (6.3)	

Complications			
Grade 0-I	61.3 (73)	69.8 (67)	0.20
Grade II–V	38.7 (46)	30.2 (29)	

Cost			
Median	21,109 (6,412–111,409)	20,157 (6,951–135,993)	0.40
Mean	23,235 (13,960)	25,210 (19,893)	

Readmission	15.1 (18)	15.1 (14)	0.99

Mortality	2.5 (3)	3.1 (3)	0.79

**Table 6:** Differences in LOS and cost by procedure type in ERAS versus non-ERAS groups, 2013–2015.

Surgery type	Mean postop LOS				Mean total cost			
	ERAS	Non-ERAS	*T* score	*p* value	ERAS	Non-ERAS	*T* score	*p* value
Pancreatectomy	9.0	9.9	−0.53	0.60	$30,524	$32,787	−0.58	0.57
Other abdominal	5.0	6.4	−1.3	0.21	$17,713	$21,268	−1.3	0.19
Colectomy	5.3	7.6	−1.9	0.059	$20,733	$24,219	−0.91	0.37
Intestinal resection	4.8	7.5	−3.5	0.001	$18,391	$20,892	−0.90	0.37
Hepatic resection	3.8	7.4	−2.9	0.0057	$16,770	$27,213	−3.2	0.0030
Gastrectomy	5.8	13.1	−2.1	0.045	$18,915	$40,853	−2.2	0.043
